# A Case of Isolated Renal Light Chain Amyloidosis With Hematuria and Low Complement Levels

**DOI:** 10.7759/cureus.38572

**Published:** 2023-05-05

**Authors:** Nismat Javed, Kalpana Uday

**Affiliations:** 1 Internal Medicine, BronxCare Health System, New York City, USA; 2 Nephrology, BronxCare Health System, New York CIty, USA; 3 Internal Medicine, Icahn School of Medicine at Mount Sinai, New York City, USA; 4 Internal Medicine, Grand Concourse Dialysis Facility, Bronx, USA

**Keywords:** clinical features, treatment, hematuria, low complement, al amyloidosis

## Abstract

The prevalence of light chain (AL) amyloidosis, characterized by the deposition of amyloid chains, is gradually increasing. The clinical features of the disease depend upon the location of amyloid deposition and can manifest in many forms. Although laboratory investigations can reveal proteinuria and change in complement levels, hematuria and low complement levels are rarely reported. There are very few cases of renal AL amyloidosis presenting as persistent hematuria. We present the case of a 54-year-old female presenting with abdominal pain, proteinuria, and moderate persistent hematuria on admission who was later diagnosed with AL amyloidosis on biopsy.

## Introduction

Light chain (AL) amyloidosis is characterized by the deposition of amyloid chains in various body organs [1]. The prevalence of AL amyloidosis in the US has increased gradually over the past few years. Estimates from 2015 revealed that the prevalence has increased from 15.5 to 40.5 cases per million, with a similar increase in incidence [2]. The renal system is commonly affected in most cases [3]. Clinical manifestations depend upon the location of amyloid deposition, with the common presentation being proteinuria to rarer forms, including nephrogenic diabetes insipidus [4]. There are very few cases of renal AL amyloidosis presenting as persistent hematuria. We present the case of a 54-year-old female presenting with abdominal pain, proteinuria, and moderate persistent hematuria on admission who was later diagnosed with AL amyloidosis on biopsy.

## Case presentation

A 54-year-old female with a past medical history of hypertension, gastroesophageal reflux disease, chronic kidney disease stage III (baseline creatinine 1.5), and IV heroin abuse presented with a two-month history of nausea, vomiting, and abdominal pain. Abdominal pain was dull, not localized, non-radiating, about 7/10 in severity, with associated non-bilious and non-bloody vomiting. She was not adherent to her anti-hypertensive medications and denied the use of non-steroidal anti-inflammatory drugs. On presentation, her vital signs revealed a temperature of 36.8 degrees C, a pulse of 101 beats/minute, blood pressure of 126/87, and saturation of 99% on room air. A physical exam revealed normocephalic, atraumatic head circumference without any obvious ocular findings. The lungs were bilaterally clear on auscultation, and normal heart sounds were present without any additional murmurs or gallops. The abdominal examination did not reveal any tenderness or organomegaly, and the kidneys were not palpable. Suprapubic fullness was also not appreciated. Pulses were bilaterally palpable in both extremities, and no overt edema was noticed. Her labs were significant for leukopenia, anemia, low bicarbonate, and elevated blood urea nitrogen-creatinine ratio (Table 1). Urinalysis revealed proteinuria (600 mg/dl), moderate persistent hematuria, few mucous cells, and moderate bacteriuria. Urine culture was negative. The lipid panel was significant for high cholesterol and low-density lipoprotein levels (Table 1).

**Table 1:** Initial laboratory investigations. HDL: High-density lipoprotein; LDL: Low-density lipoprotein.

Investigation	Result	Normal Range
Serum Hemoglobin (g/dl)	6.8	12.0-16.0
Serum WBC (/uL)	4600	4800-10800
Serum Platelets (/uL)	151000	150000-400000
Serum Sodium (mEq/L)	137	135-145
Serum Potassium (mEq/L)	4.1	3.5-5.0
Serum Calcium (mEq/L)	10.3	8.5-10.5
Serum Chloride (mEq/L)	93	98-108
Serum Glucose (mg/dl)	77	70-120
Serum Bicarbonate (mEq/L)	12	24-30
Serum Blood Urea Nitrogen (mg/dl)	149	6-20
Serum Creatinine (mg/dl)	17.4	0.5-1.5
Serum Cholesterol (mg/dl)	267	170-240
Serum HDL (mg/dl)	34	34-82
Serum LDL (mg/dl)	208	<160
Serum Triglycerides (mg/dl)	127	55-150

**Figure 1:**
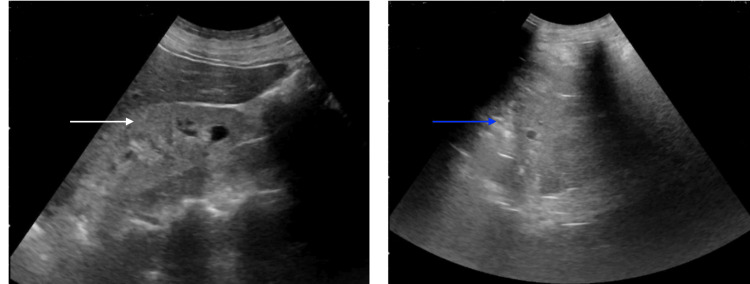
Ultrasound of the bilateral kidneys. White arrow: Right-sided kidney with echogenicities; Blue arrow: Left-sided kidney with echogenicities.

Chest X-ray was unremarkable. Ultrasound abdomen revealed small-sized echogenic kidneys (Figure 1).

**Figure 2:**
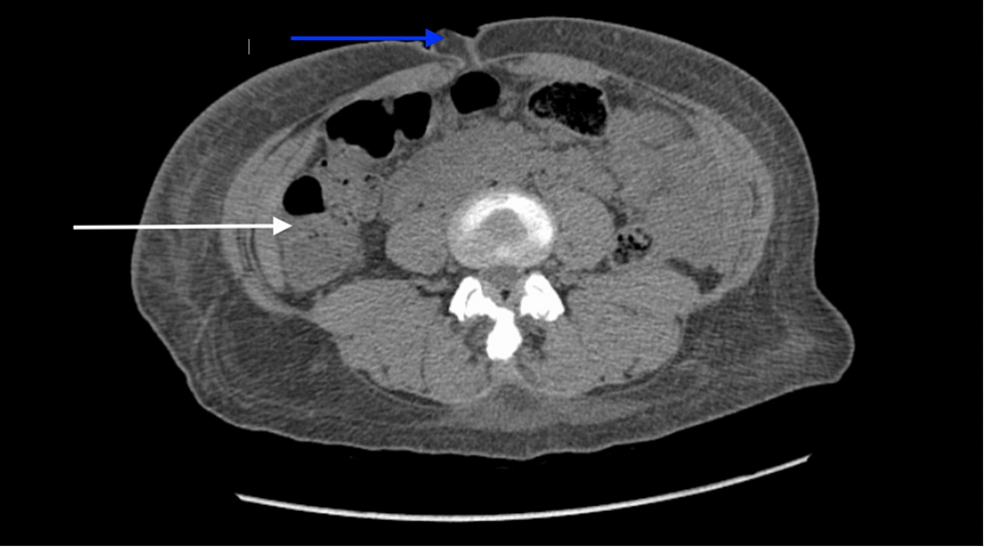
CT scan of the abdomen and pelvis without contrast. White arrow: Stool retention visualized in intestine; Blue arrow: Umbilical hernia.

CT scan of the abdomen and pelvis without contrast was significant for stool retention and umbilical hernia (Figure 2).

**Figure 3:**
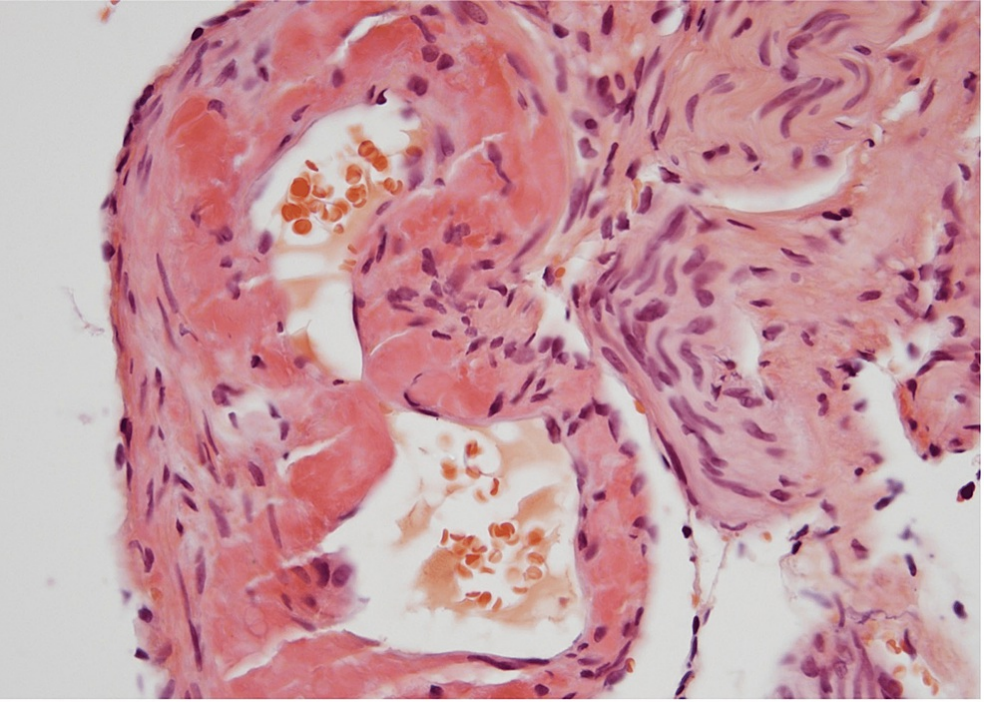
Congo red staining of the arteriole.

**Figure 4:**
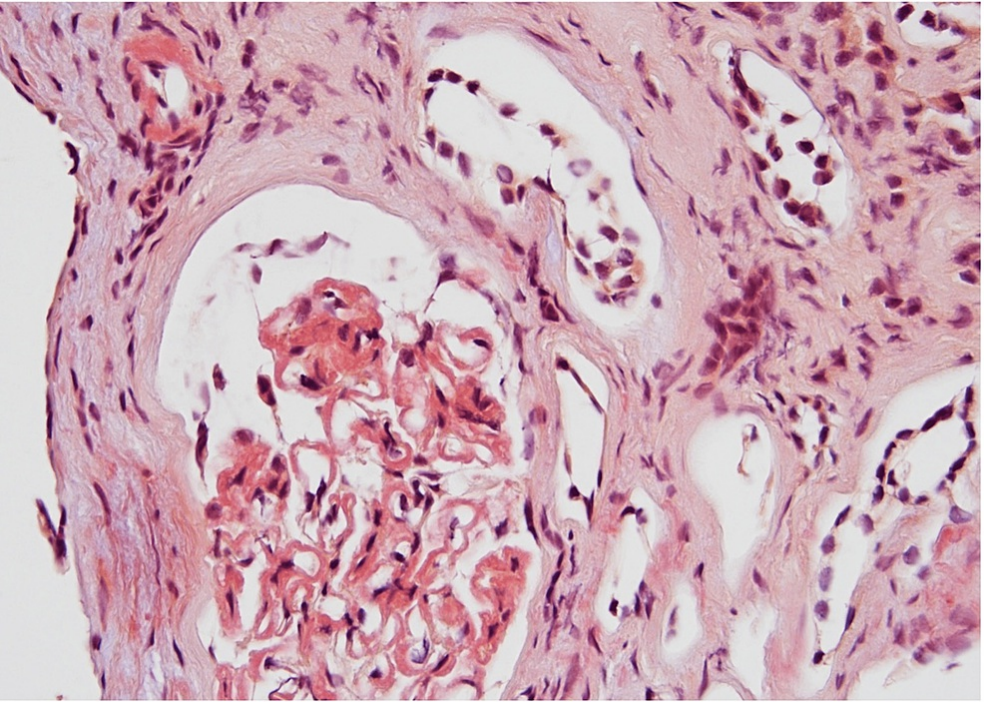
Congo red staining of glomerulus, the blood vessel (frozen section).

Possible differential diagnoses for the symptoms included acute kidney injury superimposed on chronic kidney disease, chronic glomerulonephritis, hypertensive nephropathy, and nephrotic syndrome. Nephrology was consulted, and further workup was sent. Urine electrolytes revealed fractional excretion of sodium (FENa) to be 1.0%. Autoimmune workup was negative, including antinuclear antibody (ANA), anti-dsDNA, anti-glomerular basement membrane, anti-myeloperoxidase, and anti-proteinase-3 antibodies. Cryoglobulins were not detected. Further, the workup revealed a low complement C3 level (62.0 mg/dl), positive hepatitis B core total antibody, positive hepatitis B surface antibody, negative hepatitis B core IgM antibody, and negative hepatitis B surface antigen. The urine protein/creatinine ratio was elevated (6922 mg/g). Urine electrophoresis results were suggestive of glomerular proteinuria. Urine immunofixation results were unremarkable. Serum electrophoresis and immunofixation results were unremarkable. The patient underwent hemodialysis. The patient’s symptoms, specifically nausea, vomiting, and abdominal pain, improved, and creatinine levels decreased to 2.6 mg/dl. However, her creatinine levels continued to rise. A kidney biopsy was performed for diagnostic purposes considering the unclear etiology of the acute kidney injury. The biopsy sample contained eight glomeruli, out of which five were globally sclerotic. The glomeruli exhibited segmental to global mesangial matrix expansion. No significant endocapillary proliferation was noted. Congo red staining was positive in glomeruli and arterioles with green birefringence on polarization (Figures 3-4).

**Figure 5:**
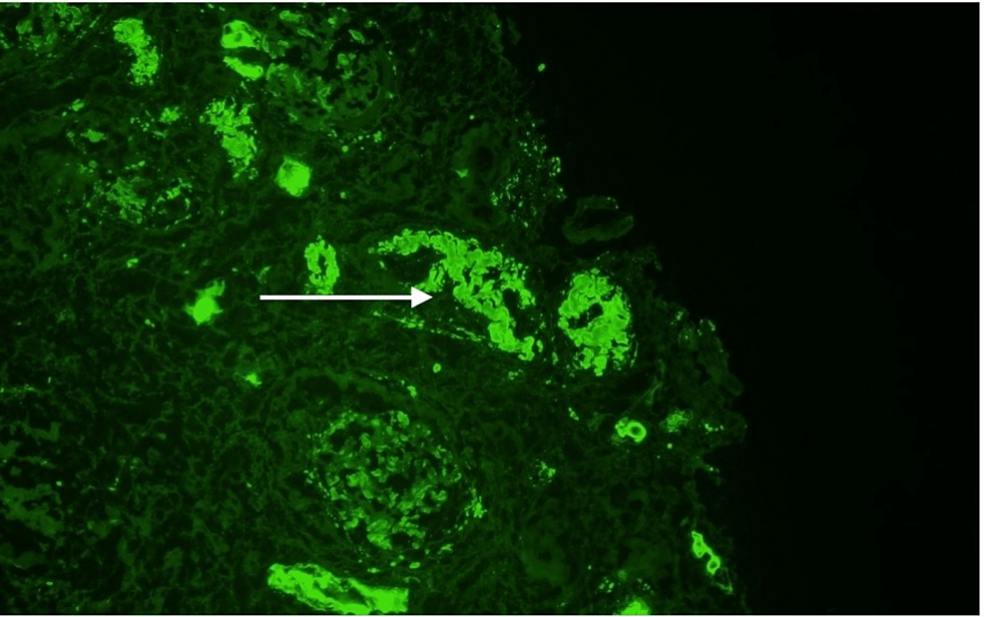
Immunofluorescence for lambda. White arrow: Area of immunofluorescence visualized.

**Figure 6:**
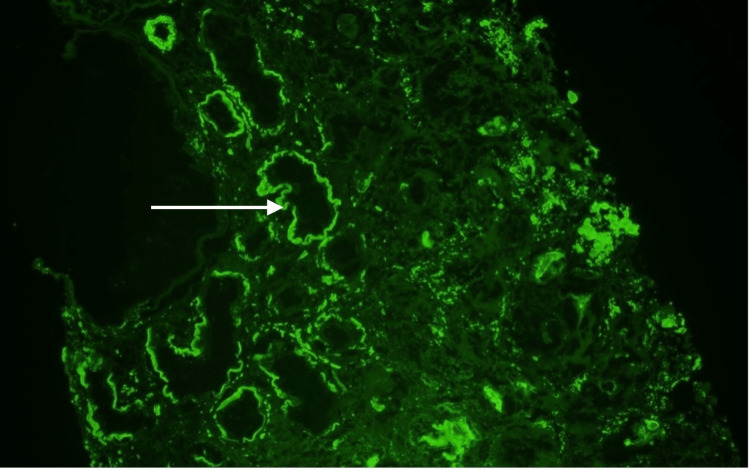
Immunofluorescence for lambda along tubular basement membranes. White arrow: Area of immunofluorescence visualized.

**Figure 7:**
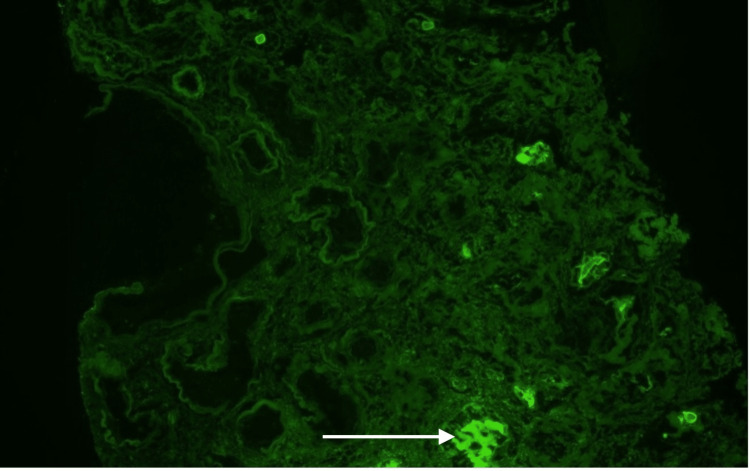
Immunofluorescence for kappa along tubular basement membranes. White arrow: Area of immunofluorescence visualized.

Microscopic results from the biopsy revealed moderately severe tubulointerstitial scarring accompanied by largely mononuclear interstitial inflammation. Proximal tubules exhibited diffuse degenerative changes. Severe arteriosclerosis and moderate arteriolosclerosis were noted. Immunofluorescence findings of 3+ linear staining for lambda along tubular basement membranes (Figures 5-6) with negative staining for kappa (Figure 7) raised the possibility of concurrent lambda-AL deposition disease.

However, no powdery electron-dense deposits were seen along the basement membrane, ruling out AL deposition disease. These features were diagnostic of renal AL amyloidosis type with a background of severe chronic changes. Hematology was consulted. Hematologic workup, including skeletal survey, was only significant for elevated beta 2 microglobulin level (>8 g/dl). The patient subsequently underwent permanent dialysis catheter placement and was discharged. Currently, she has an outpatient follow-up with hematology for further management of AL amyloidosis. Her renal function did not improve, and she is regularly following up at a long-term dialysis center unit.

## Discussion

Hematuria as a presenting feature of isolated renal AL amyloidosis is relatively uncommon. To date, there are seven reports discussing patients with hematuria who were later diagnosed with either isolated or systemic amyloidosis [5-10]. Of these seven reports, three articles discussed hematuria as a presenting feature of AL amyloidosis [5, 7, 8]. Renal AL amyloidosis was usually diagnosed in younger individuals ranging from 30 to 40 years of age [6,9], compared to the 54-year-old patient being discussed. Although, in our case, the patient had a prior history of chronic kidney disease and drug use, these risk factors would predispose the patient to nephrotic range proteinuria and not specifically AL amyloidosis [11]. However, a few cases of transthyretin amyloid (ATTR)-associated amyloidosis have been documented in older individuals presenting with systemic manifestations [8,10]. Isolated renal AL amyloidosis was mostly diagnosed in females [6]. The presenting symptom in our study was abdominal pain; however, patients in previous reports also presented with generalized weakness [6], night sweats [6], rashes [6], hematuria [5, 7, 8, 9], and multiorgan failure [8]. Hematuria was most commonly found in cases with senile wild-type systemic amyloidosis [8], transthyretin amyloidosis of the urinary bladder [10], AA amyloidosis [6], and amyloidosis with concomitant crescentic glomerulonephritis [7]. In previous studies, proteinuria and albumin creatinine ratios usually ranged around 1-2 g/day [6, 9]. However, in our case, proteinuria was significantly higher, and the patient was stable hemodynamically. However, Jayakrishnan T et al. [8] and Mihout F et al. [9] detailed hemodynamic instability at the time of presentation. Autoimmune workup in most previous cases was unremarkable consistent with the results of our report.

Serum beta-2-microglobulin levels were high in most cases, as observed in our patient. Electrophoresis results in such a cohort of patients showed either glomerular proteinuria [6], as seen in our patient, or no abnormalities [9]. Zakharova E et al. [6] discussed normal C3 complement levels in one case, whereas our patient had low complement levels. The differential diagnoses for renal diseases with low complement levels include post-infectious glomerulonephritis, infectious endocarditis, systemic lupus erythematosus, membranoproliferative glomerulonephritis, C3 glomerulopathy, cryoglobulinemia, and atheroembolic renal disease [12]. Therefore, a biopsy, as performed in most cases reviewed, is one of the essential tools needed for diagnosis and clinical decision-making. In our case, the biopsy did not reveal a cause for the low complement levels. Management options for systemic AL amyloidosis include steroids and extensive chemotherapy regimens, including bortezomib and melphalan. However, options for isolated renal AL amyloidosis are fairly limited. In this case, the patient did not exhibit any systemic signs of the disease and was, therefore, managed conservatively with hemodialysis, watchful waiting, and close follow-up. 

## Conclusions

Renal AL amyloidosis is a relatively rare disease with a varying clinicopathological spectrum. The demographic characteristics associated with the disease also vary. Although systemic manifestations with other disorders are fairly common, there is limited data about the isolated renal variant of AL amyloidosis. Furthermore, treatment primarily targets the systemic variations of amyloidosis, and very limited evidence is available regarding therapies in renal AL amyloidosis. Therefore, further studies have to be performed to evaluate this cohort of patients for better optimization of patient care.
